# Risk Behaviors for Contact Lens–Related Eye Infections Among Adults and Adolescents — United States, 2016

**DOI:** 10.15585/mmwr.mm6632a2

**Published:** 2017-08-18

**Authors:** Jennifer R. Cope, Sarah A. Collier, Hannah Nethercut, Jefferson M. Jones, Kirsten Yates, Jonathan S. Yoder

**Affiliations:** 1 Division of Foodborne, Waterborne, and Environmental Diseases, National Center for Emerging and Zoonotic Infectious Diseases, CDC.

Contact lens–related eye infections, which can lead to serious outcomes, including blindness, are associated with several risk factors, including sleeping in lenses, exposing lenses to water, not adhering to replacement schedules, and reusing disinfecting solution (*1*). In some studies, adolescent and young adult contact lens wearers have been reported to be more likely than older adult contact lens wearers to develop eye infections (*2*,*3*) and more likely to have poor contact lens hygiene practices (*2*). In 2015, CDC reported the number and demographics of adult contact lens wearers in the United States to define the population at risk for contact lens–related eye infections (*4*); however, this estimate did not include adolescents. To better understand this group of younger contact lens wearers and guide prevention efforts, a population-based survey was used to assess contact lens wear, care behaviors, risk factors, and demographics among persons aged 12–17 years (referred to as adolescents in this report), young adults aged 18–24 years, and older adults aged ≥25 years in the United States. In 2016, an estimated 3.6 million adolescents (14.5%) wore contact lenses. Of the adolescents who wore contact lenses, 85% reported at least one behavior that put them at risk for a contact lens–related eye infection, compared with 81% of young adults, and 88% of older adults. These findings can inform the creation of age-specific targeted prevention messages aimed at contact lens wearers and establish a baseline for evaluating trends in contact lens wear, care habits, and contact lens–related risk behaviors.

To describe contact lens wear and care behaviors, risk factors, and demographics for adolescents and adults in the United States, the Porter Novelli 2016 summer HealthStyles and YouthStyles survey, an online survey of 4,548 U.S. adults (aged ≥18 years) and 1,618 U.S. adolescents (aged 12–17 years) was used. Adolescent participants lived in the households of the adult participants.* The 2016 Porter Novelli Styles survey participants were part of the GfK KnowledgePanel, a nationally representative online panel with members recruited through probability-based sampling by postal address. Computer and Internet access were provided to complete the survey where needed. For completing this survey and others, households received rewards points, which they could redeem for prizes generally worth less than $500. The sample was weighted on nine factors (sex, age, household income, race/ethnicity, household size, education, census region, metropolitan status, and prior Internet access) to match the Current Population Survey conducted by the U.S. Census Bureau. Participants were asked to provide demographic and contact lens wearing information. If they wore contact lenses, they were asked about contact lens hygiene behaviors and risk factors associated with contact lens–related eye infections. The question regarding contact lens hygiene behaviors was “When you wear contact lenses, which of these actions do you do on a regular basis (sometimes, most of the time, or always)?”

In 2016, an estimated 3.6 million adolescents aged 12–17 years (14.5% of adolescents), 7.5 million young adults aged 18–24 years (24.4% of young adults), and 33.9 million older adults aged ≥25 years (15.5% of adults) in the United States wore contact lenses. Among lens wearers, 90.4% of adults and 87.8% of adolescents reported wearing soft contact lenses (lenses made of soft, flexible plastics that allow oxygen to pass through to the cornea). No significant demographic differences between adolescent contact lens wearers and adolescent nonwearers were observed (Table 1). By race, older adult lens wearers were more likely to be white than were adolescent lens wearers. No significant geographic region or metropolitan residency differences were observed for either adolescents or adults, regardless of lens-wearing status.

**TABLE 1:** Demographic characteristics of adolescent contact lens wearers (aged 12–17 years), by type of contact lens, compared with adolescent nonwearers, young adult lens wearers (aged 18–24 years), and older adult lens wearers (aged ≥25 years) — United States, 2016*

Characteristic	% (95% CI)					
	Adolescent soft CL wearers (n = 119)	Adolescent gas permeable or other^†^ CL wearers (n = 16)	All adolescent CL wearers^§^ (n = 135)	Adolescent nonwearers (n = 810)	Young adult CL wearers (n = 124)	Older adult CL wearers (n = 571)
**Sex**						
Female	52.6 (41.6–63.5)	48.2 (11.8–84.5)	52.3 (41.6–62.9)	48.8 (44.7–52.8)	69.3 (56.8–81.8)	65.2 (60.4–70.0)
Male	47.4 (36.5–58.4)	51.8 (15.5–88.2)	47.7 (37.1–58.4)	51.2 (47.2–55.3)	30.7 (18.2–43.2)	34.8 (30.0–39.6)
**Race/Ethnicity**						
White, non-Hispanic	49.9 (39.0–60.7)	42.3 (6.7–77.9)	48.4 (37.9–58.9)	55.0 (50.9–59.2)	56.7 (42.7–70.6)	66.9 (61.8–71.9)
Hispanic	26.6 (16.1–37.1)	15.7 (0.0–34.4)	25.6 (15.8–35.4)	22.4 (18.5–26.2)	21.1 (9.2–33.0)	11.4 (8.3–14.5)
Black, non-Hispanic	12.0 (3.4–20.6)	42.0 (4.9–79.2)	15.8 (6.4–25.2)	13.5 (10.4–16.6)	7.1 (0.6–13.6)	10.3 (7.2–13.4)
Other or multiracial	11.5 (3.9–19.2)	—	10.3 (3.4–17.2)	9.1 (6.7–11.5)	15.2 (3.3–27.0)	10.7 (6.5–14.9)
**Metropolitan living area**						
Metro	90.5 (84.8–96.3)	56.9 (18.5–95.3)	86.3 (78.1–94.5)	85.0 (81.8–88.1)	93.6 (86.2–100.0)	86.2 (82.7–89.7)
Nonmetro	9.5 (3.7–15.2)	43.1 (4.7–81.5)	13.7 (5.5–21.9)	15.0 (11.9–18.2)	6.4 (0.0–13.8)	13.8 (10.3–17.3)
**Geographic region**						
Northeast	16.2 (8.0–24.5)	11.6 (0.0–28.7)	15.9 (8.2–23.5)	16.9 (13.9–19.8)	27.5 (15.7–39.2)	17.7 (14.0–21.3)
Midwest	27.4 (18.9–35.9)	22.5 (0.0–50.8)	26.8 (18.5–35.1)	20.4 (17.3–23.4)	24.3 (12.7–35.9)	23.5 (19.4–27.5)
South	36.5 (25.8–47.2)	54.7 (19.8–89.7)	38.3 (27.6–48.9)	37.7 (33.7–41.7)	33.8 (20.6–47.0)	35.6 (30.9–40.4)
West	19.9 (10.5–29.3)	11.2 (0.0–27.1)	19.1 (10.4–27.7)	25.0 (21.3–28.7)	14.4 (4.3–24.6)	23.2 (18.9–27.5)

**Abbreviations:** CI = confidence interval; CL = contact lens.

* Based on responses to Porter Novelli 2016 summer HealthStyles and YouthStyles surveys with questions on contact lens use and wearer/nonwearer demographics.

^†^ Other indicates contact lens wearers who said they wore a type of contact lens not included among the survey choices.

^§^ Some individual columns do not sum to 100.0 because of rounding.

At least one contact lens hygiene risk behavior was reported by older adult (87.5%), young adult (80.9%), and adolescent (85.3%) lens wearers (Table 2). The most frequently reported risk behaviors in adolescents were not visiting an eye doctor as least annually, sleeping or napping in lenses, and swimming in lenses (Table 2). Among young adults and older adults, the most frequently reported risk behaviors were replacing lenses at intervals longer than those prescribed, replacing lens storage cases at intervals longer than those recommended, swimming in lenses, and sleeping or napping in lenses. Adolescents were significantly less likely to report replacing lenses at intervals longer than prescribed and replacing lens storage cases at intervals longer than recommended. Although both adults and adolescents most commonly reported purchasing contact lenses through their eye care provider, both young adults and older adults were more likely than adolescents to purchase lenses on the Internet. A higher percentage of young adults (14.6%, 1.1 million) and older adults (11.4%, 3.9 million) than adolescents (4.2%, 152,000) reported ever experiencing a red or painful eye that required an eye care provider visit.

**TABLE 2:** Prevalence of risk behaviors* for contact lens–related eye infections and outcomes among adolescent (aged 12–17 years), young adult (aged 18–24 years), and older adult (aged ≥25 years) contact lens wearers — United States, 2016

Characteristic	% (95% CI)		
	Adolescent CL wearers	Young adult CL wearers	Older adult CL wearers
**Risk factor/Behavior**			
Sleeping or napping in CLs	29.8 (19.7–40.0)	33.3 (20.9–45.7)	32.9 (28.3–37.5)
Topping off solution^†^	10.6 (4.9–16.2)	19.1 (8.4–29.8)	11.0 (7.8–14.3)
Replacing lenses at intervals longer than prescribed	23.7^§,¶^ (14.7–32.6)	52.4 (38.8–66.1)	44.5 (39.7–49.4)
Did not visit eye doctor at least annually	43.9 (33.1–54.6)	24.0 (11.8–36.1)	29.6 (25.0–34.3)
Replacing CL case at interval longer than recommended	22.8^§,¶^ (14.5–31.2)	40.5 (27.2–53.7)	41.7 (36.9–46.5)
Storing lenses in tap water	9.5^¶^ (3.3–15.7)	11.0 (2.1–19.9)	2.3 (0.8–3.8)
Rinsing lenses in tap water	7.1 (2.7–11.5)	12.1 (3.2–21.0)	6.2 (4.2–8.2)
Swimming in CLs	27.2 (18.4–36.0)	28.1 (16.3–40.0)	33.2 (28.7–37.7)
Any risk behavior	85.3 (78.7–91.9)	80.9 (70.0–91.8)	87.5 (84.2–90.7)
**Source of purchase**			
Eye care provider office	68.0 (58.2–77.9)	65.5 (52.7–78.4)	65.4 (60.6–70.2)
Retail store without eye exam	15.8 (9.4–22.2)	22.5 (11.4–33.7)	21.3 (17.1–25.5)
Internet	10.5 (5.1–15.8)	20.6 (9.6–31.5)	18.8 (14.9–22.6)
Other	3.6 (0.0–7.4)	—	1.7 (0.5–3.0)
**Ever had a red/painful eye while wearing CLs that required a doctor visit**	4.2 (0.7–7.8)	14.6 (5.1–24.1)	11.4 (8.1–14.8)

**Abbreviations:** CI = confidence intervals; CL = contact lens.

*As assessed by the question “When you wear contact lenses, which of these actions do you do on a regular basis (sometimes, most of the time, or always)?”

^†^ Adding new solution to existing solution in the contact lens case instead of emptying and cleaning the case before adding new solution.

^§^ p-value <0.05 compared with young adult CL wearers.

^¶^ p-value <0.05 compared with older adult CL wearers.

## Discussion

An estimated one in seven adolescents and one in six adults in the United States wore contact lenses in 2016, and approximately six of seven lens wearers reported at least one behavior putting them at risk for a serious contact lens–related eye infection. Lens wearers most commonly reported sleeping or napping in lenses, swimming in lenses, and replacing both lenses and lens storage cases at intervals longer than those recommended.

A previous study suggested that adolescents and young adults have lower compliance with contact lens hygiene recommendations and have a greater risk for corneal inflammatory events, a category of eye problems that includes serious eye infections (*3*). Young adults in this survey were significantly more likely to replace lenses and cases at intervals longer than those recommended than were adolescents. These findings might reflect the fact that most adolescents are still living with their parents who might help to reinforce good contact lens hygiene practices whereas young adults might have recently left home and are no longer subject to parental reminders (*2*). Young adults also might have fewer resources (e.g., money and transportation) to regularly visit eye care providers and obtain hygiene education or regularly replace contact lenses, lens storage cases, and solution (*3*). Young adults have been reported to have poor planning and a more impulsive lifestyle in relation to contact lens hygiene, possibly related to crowded living conditions (e.g., dormitories, living with roommates, and sharing bathrooms), alcohol consumption, and attitudes conducive to taking greater risks (*2*). A higher percentage of young adults also reported ever having a red or painful eye while wearing contact lenses, suggesting that poor hygiene practices might lead to complications.

Engaging in risky contact lens behaviors can lead to potentially serious eye infections (*1*). Substantial percentages of adults and adolescents reported noncompliance with recommended contact lens storage case and lens replacement schedules. Infrequent contact lens storage case replacement has been associated with microbial keratitis (*5*), and lens wearers who do not replace their lenses as often as recommended report more complications and eye discomfort (*6*). Not replacing contact lenses and contact lens storage cases as often as recommended increases the risk for contact lens–related eye infections because recurrent handling of the contact lenses and storage cases presents the opportunity to introduce microorganisms; in addition, the moist surfaces of the lens and storage case provide an environment favorable to microbial growth (*7*).

Exposing contact lenses to water through swimming or showering increases the risk for infection because microorganisms living in water can be transferred to the eye. Even household tap water, although safe for drinking, contains microorganisms that can contaminate lens cases and contact lenses and cause eye infections, especially when not replaced at recommended intervals (*8*). Sleeping in contact lenses was another commonly reported risk behavior. Although some soft and rigid contact lenses have Food and Drug Administration approval for overnight wear, sleeping in any type of contact lens increases the risk for eye infections (*9*).

The findings in this report are subject to at least five limitations. First, respondents were part of a larger survey that was not specifically focused on contact lens behaviors. Therefore, participants might not have been representative of contact lens wearers in the United States. Second, adolescents were sampled through convenience sampling, specifically those living in the household of an adult taking the larger survey. This sampling method led to a small sample size of adolescent respondents. In addition, the number of young adults in the sample was small. Third, the sampling method differed from a sample in a previous report (*4*) that also asked risk behavior questions in a different manner (i.e., “ever” versus “regular” behaviors) and produced differences in the percentage of respondents reporting outcomes and behaviors. Fourth, because data were self-reported, respondents might have been reluctant to report risk behaviors because of social desirability bias. Finally, for the contact lens hygiene and outcomes questions, no period was stipulated; this might affect the comparison among age groups because the duration of contact lens use might differ and individual practices can change over time.

Although adolescent contact lens wearers reported engaging in some healthier contact lens hygiene behaviors than their adult counterparts, there is still room for improvement to prevent potentially serious outcomes, including blindness. Prevention efforts should focus on encouraging contact lens wearers to replace their contact lens storage case regularly and to avoid sleeping or napping in contact lenses. There are insufficient data regarding the appropriate frequency of lens case and contact lens replacement, but contact lens wearers who do not follow recommended lens replacement schedules have more complications and self-reported discomfort than contact lens wearers who follow the replacement recommendations (*6*).^†^

 Existing health communication strategies known to influence behavior change in adolescents (e.g., appeals to vanity and social norms marketing) can be applied to communication efforts focusing on contact lens hygiene behaviors in this population (*10*). Additionally, encouraging adolescents to adopt healthy contact lens wear and care habits early might help them maintain these habits into young adulthood, when the frequency of reported risk behaviors increases. Prevention messages targeting young adults can be shaped around the lifestyle changes known to occur in this population.

**Summary**

**What is already known about this topic?**

In 2015, CDC established that there were approximately 41 million contact lens wearers aged ≥18 years in the United States, the majority of whom engaged in behaviors that put them at risk for serious eye infections.

**What is added by this report?**

In 2016, there were an estimated 3.6 million adolescents aged 12–17 years in the United States who wore contact lenses. Of the adolescents who wore contact lenses, 85% reported at least one behavior that put them at risk for a contact lens–related eye infection, compared with 81% of young adults, and 88% of older adults.

**What are the implications for public health practice?**

Although adolescent contact lens wearers engage in some healthier contact lens hygiene behaviors than do their adult counterparts, there is room for improvement in order to prevent potentially serious outcomes including blindness. Prevention efforts should focus on encouraging contact lens wearers to replace their contact lens storage case regularly and to avoid sleeping or napping in contact lenses.

## Footnotes

* Porter Novelli Public Services. Summer HealthStyles and YouthStyles 2016 methodology; Washington, DC.
